# A Conserved Motif in the ITK PH-Domain Is Required for Phosphoinositide Binding and TCR Signaling but Dispensable for Adaptor Protein Interactions

**DOI:** 10.1371/journal.pone.0045158

**Published:** 2012-09-18

**Authors:** Nupura Hirve, Roman M. Levytskyy, Stephanie Rigaud, David M. Guimond, Tomasz Zal, Karsten Sauer, Constantine D. Tsoukas

**Affiliations:** 1 Molecular Biology Institute and Center for Microbial Sciences, Department of Biology, San Diego State University, San Diego, California, United States of America; 2 Department of Immunology and Microbial Science, The Scripps Research Institute, La Jolla, California, United States of America; 3 MD Anderson Cancer Center, Department of Immunology, The University of Texas, Houston, Texas, United States of America; J. Heyrovsky Institute of Physical Chemistry, Republic of Czech

## Abstract

Binding of the membrane phospholipid phosphatidylinositol 3,4,5-trisphosphate (PIP_3_) to the Pleckstrin Homology (PH) domain of the *Tec* family protein tyrosine kinase, Inducible T cell Kinase (ITK), is critical for the recruitment of the kinase to the plasma membrane and its co-localization with the TCR-CD3 molecular complex. Three aromatic residues, termed the FYF motif, located in the inner walls of the phospholipid-binding pocket of the ITK PH domain, are conserved in the PH domains of all *Tec* kinases, but not in other PH-domain containing proteins, suggesting an important function of the FYF motif in the *Tec* kinase family. However, the biological significance of the FYF amino acid motif in the ITK-PH domain is unknown. To elucidate it, we have tested the effects of a FYF triple mutant (F26S, Y90F, F92S), henceforth termed FYF-ITK mutant, on ITK function. We found that FYF triple mutation inhibits the TCR-induced production of IL-4 by impairing ITK binding to PIP_3_, reducing ITK membrane recruitment, inducing conformational changes at the T cell-APC contact site, and compromising phosphorylation of ITK and subsequent phosphorylation of PLCγ_1_. Interestingly, however, the FYF motif is dispensable for the interaction of ITK with two of its signaling partners, SLP-76 and LAT. Thus, the FYF mutation uncouples PIP_3_-mediated ITK membrane recruitment from the interactions of the kinase with key components of the TCR signalosome and abrogates ITK function in T cells.

## Introduction

The *Tec* family of non-receptor protein tyrosine kinases is important for hematopoietic cell development and function [1]. The *Tec* family member, Inducible T cell Kinase (ITK), regulates TCR-induced signaling events including intracellular Ca^++^ mobilization, actin polymerization, and the transcriptional activation of Th_2_ cytokine genes (reviewed in ref. [2]).

ITK is organized in structural domains that play critical roles in the regulation of its functions [3]. The most N-terminally located domain of ITK, the Pleckstrin Homology (PH) domain, mediates TCR-induced recruitment and localization of the kinase to the plasma membrane [4], [5]. Production and turnover of the membrane phospholipid phosphatidylinositol 3,4,5-trisphosphate (PIP_3_) and its interaction with the PH domain are critical for ITK recruitment to the plasma membrane and its co-localization with the TCR-CD3 molecular complex [4]–[6]. We recently demonstrated that the soluble ligand inositol 1,3,4,5 tetrakisphosphate (IP_4_) plays a critical role in regulating ITK-PH domain interactions with the plasma membrane [5].

About 300 eukaryotic proteins contain PH domains [7]. Although these PH domains share limited sequence homology, they display very high structural similarity that consists of two perpendicular β sheets with an α helix at the C-terminal end [8]. Three aromatic residues termed the FYF motif, located in the inner walls of the phospholipid-binding pocket, are practically invariant in the PH domains of all *Tec* kinases. The fact that these three amino acids are not conserved in other PH-domains suggests that this motif might be involved in a specific function of *Tec* kinases [9]. It has been suggested that in the B lymphocyte expressed *Tec* kinase BTK, the amino acids of the FYF motif (F25, Y112, F114) might be critical for membrane phospholipid binding and their mutation is suspected to cause X-linked agammaglobulinemia [9].

The FYF motif is also found in the ITK-PH domain (F26, Y90, F92), but its function in ITK is unknown. To elucidate it, we have tested the effects of FYF motif triple mutation (F26S, Y90F, F92S), henceforth termed FYF-ITK mutant, on ITK function in T cells. Collectively, the data presented here indicate that the aromatic amino acids comprising the FYF motif are important for ITK-dependent cytokine production through a mechanism that depends on the TCR-induced recruitment of ITK to the cell membrane where it interacts with phospholipids and becomes activated for the delivery of downstream signals. Interestingly, the FYF motif is dispensable for the interaction of ITK with two of its signaling partners, SLP-76 and LAT, which in the past have been shown to be critical for the TCR-induced activation of ITK (reviewed in ref. [2]). Thus, the FYF mutation uncouples PIP_3_-mediated ITK membrane recruitment from the interactions of the kinase with key components of the TCR signalosome and abrogates ITK function in T cells.

## Results

### Deficient IL-4 Production by FYF-ITK Mutant Nucleofected T Cells

**Figure 1:**
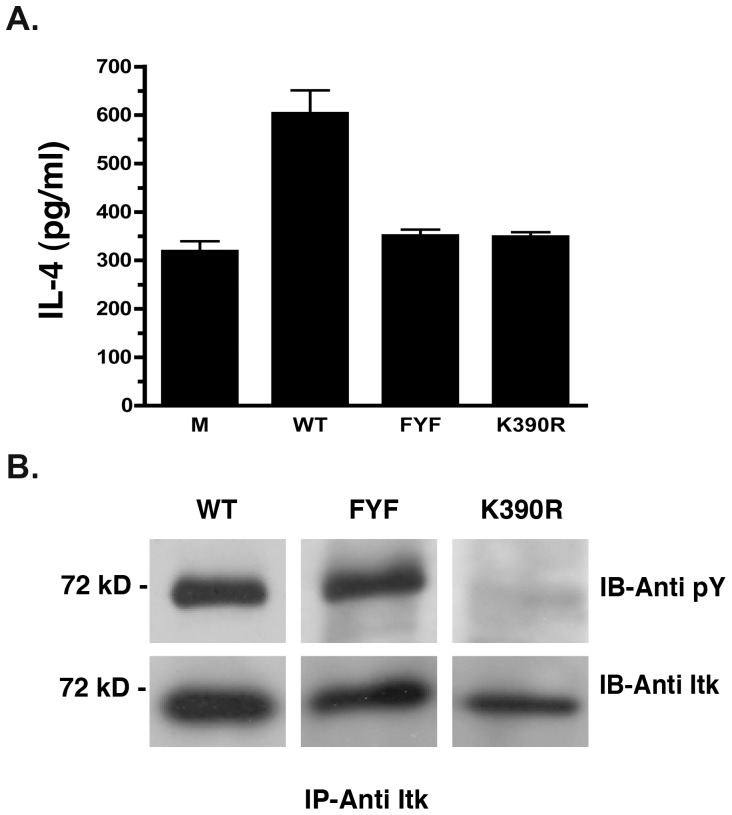
Deficient IL-4 production by FYF-ITK mutant nucleofected T cells. *(A),* Culture supernatants of murine *ITK* ^−/−^ thymocytes that had been nucleofected with the indicated GFP-ITK cDNA constructs or mock nucleofected (M) and stimulated under Th_2_ skewing conditions (as described in Materials and Methods) were assessed for their IL-4 content by ELISA. Results are displayed as the averages (±SEM) of three replicate experiments. Non-stimulated culture supernatants of WT-ITK nucleofected cells contained an average of 69 pg IL-4 per ml. The WT-ITK values are significantly different from either K390R or FYF at p<0.05 as determined by the student’s t test. The expression of the GFP-ITK constructs was comparable in all experimental groups. Average GFP-MFI was 1509 for WT-ITK, 1655 for FYF-ITK, and 1325 for K390R-ITK. *(B),* Anti-ITK immune complexes from lysates of HEK-293T cells that had been transfected with the indicated untagged ITK constructs and then subjected to *in vitro* kinase assay were resolved by SDS-PAGE followed by sequential immunoblotting with anti-pY and anti-ITK antibodies. Data are representative of two replicate experiments.

ITK is known to regulate the transcriptional activation and production of Th_2_ cytokines [10]. To determine whether FYF-ITK mutant has any effects on this function, we assessed the production of the Th_2_ signature cytokine IL-4 by ITK^−/−^ murine thymocytes that had been nucleofected with FYF-ITK mutant. In order to increase the relative frequency of IL-4 producing lymphocytes, we first skewed the cultures towards Th_2_ cytokine production, as described in the Materials and Methods section, and then stimulated them through the TCR. Compared to WT-ITK nucleofected cells, FYF-ITK mutant expressing cells produced significantly lower amounts of IL-4 (Fig. 1A). The reduction in IL-4 was equivalent to that seen with K390R-ITK nucleofected cells (Fig. 1A) that were used as negative controls since this mutation incapacitates the enzymatic activity of ITK (Fig.1B and ref. [11]). Notably, in spite of the inability of FYF-ITK mutant to support IL-4 production, this ITK mutant displayed normal enzymatic activity as assessed in an *in vitro* kinase assay (Fig. 1B). The possibility that the signal seen with FYF-ITK mutant may not be a reflection of enzymatic activity, but rather due to a co-precipitated *Src* kinase in HEK-293T cells that might have phosphorylated the FYF mutant on Y511 or Y180 is very unlikely because if this were the case, we would have also seen a signal in the K390R mutant. It is interesting that despite the forty percent reduction in IL-4 production we saw with FYF-ITK mutant transfectants, the amount of the cytokine in the culture supernatants was not completely abolished (Fig. 1A). This was also true for the K390R-ITK mutant (Fig. 1A). The possibility that this amount of IL-4 might be due to contaminating cytokine carried over from the skewing protocol is very unlikely because the cells were washed thoroughly before stimulation and non-stimulated cultures contained on the average only 69 pg/ml. However, one possible explanation is that even though the T cells used in these experiments lacked ITK, other *Tec* kinases (e.g. TEC, RLK) are present, and thus they may be contributing to the production of IL-4 in the culture supernatants [12]. Evidence to support this interpretation is that cultures of mock-nucleofected ITK-deficient thymocytes produced IL-4 in quantities equivalent to those seen in the FYF- and K390R-ITK nucleofectant cultures (Fig. 1A). Therefore, we interpret the data of these experiments to reflect a specific ITK effect.

### FYF-ITK Mutant Nucleofected Cells Display Defective PLCγ_1_ Phosphorylation

**Figure 2:**
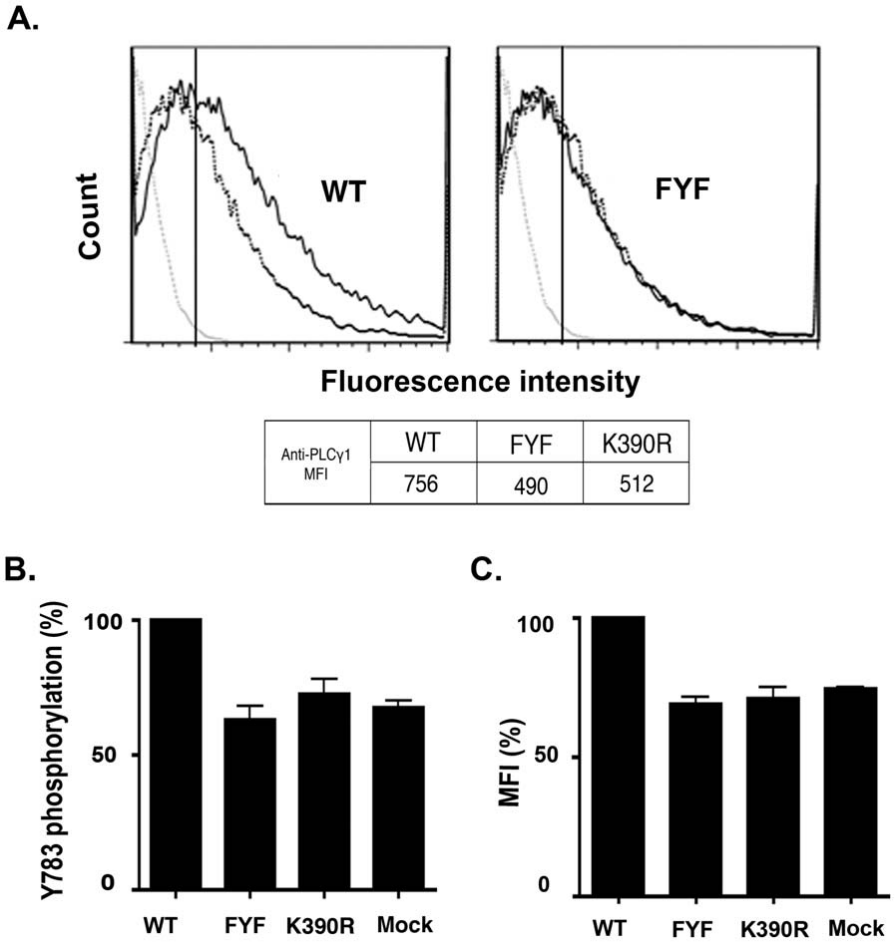
FYF-ITK mutant nucleofected cells display defective PLCγ1 phosphorylation. *(A)*, Thymocytes isolated from ITK^−/−^ mice and nucleofected with cDNA constructs encoding GFP-tagged WT-, FYF-, or K390R-ITK fusion proteins, or mock-nucleofected were stimulated or not with anti-mouse CD3ε antibodies and then analyzed by flow cytometry using Alexa 647-conjugated anti-PLCγ1 pY783 antibodies as described in the Materials and Methods section. Results are displayed as cell number versus fluorescence intensity. In each panel the grey dotted line histograms represent mock-nucleofected cells that were not stimulated as negative controls for setting an electronic gate (vertical line) for calculation of positive cells. Histograms of non-stimulated, nucleofected or mock-nucleofected cells were similar. The black dotted line histograms represent K390R-ITK nucleofected cells that were stimulated. The solid black line histograms represent WT-ITK nucleofected (left panel) and FYF-ITK mutant nucleofected (right panel) cells that were stimulated. The table inset lists anti-PLCγ1 MFI of the displayed stimulated cell histograms. *(B),* Average (±SEM) percentage of pY783 positive cells of five replicate experiments (except K390R; two replicate experiments) performed and analyzed as in panel A, and normalized as percentage of the respective WT control (average±SEM 39.6±8.7%), as described in Materials and Methods section. *(C)* Average (±SEM) anti-PLCγ1 MFI of same experiments normalized as in panel B. WT control average MFI (±SEM) 616±130. The percentages of Y783 phosphorylation and MFI in FYF-mutant and K390R-ITK nucleofected cells are significantly lower from WT-ITK at p<0.05 determined by the student’s t test. No significant differences from Mock transfected controls. The expression of the GFP-ITK constructs was comparable in all experimental groups. Average (±SEM) GFP fluorescence MFI was 1508±48 for WT-ITK, 1590±63 for FYF-ITK mutant, and 1274±104 for K390R-ITK.

The production of IL-4 and other cytokines is dependent on the activation of PLCγ_1_ [13]. Since one step for the activation of PLCγ_1_ entails the phosphorylation of its Tyr 783 by ITK [14], we wished to determine the ability of FYF-ITK mutant to phosphorylate PLCγ_1_ on this residue. To this end, we nucleofected ITK^−/−^ thymocytes with WT-, K390R-, or FYF-mutant ITK and compared the degree of TCR-induced PLCγ_1_ phosphorylation by intracytoplasmic flow cytometry using anti-pY783 specific antibodies. By assessing MFI of the staining anti-PLC γ_1_ antibody, we see 35% reduction in PLCγ_1_ phosphorylation in cells expressing FYF-ITK as compared to WT-ITK transfectants (Fig. 2A, table inset). This reduction was similar when percentage positive cells were compared (Fig. 2A, histograms). Furthermore, this decrease was similar to that seen with cells nucleofected with the kinase-dead K390R-ITK (Fig. 2A, histogram and table inset). These results were reproduced in five replicate experiments where FYF-ITK mutant and WT-ITK were compared both by percentage and MFI measurements of PLCγ_1_ phosphorylation (Fig. 2 B and C). It is interesting that similar to the observation with IL-4 production above, the phosphorylation of PLCγ_1_ was not completely abolished in either FYF-mutant or K390R-nucleofected cells. Again, we interpret this lack of complete abrogation to the presence of other *Tec* kinases in the ITK-deficient cells that may be partially redundant with ITK. This interpretation is supported by controls shown in Figure 2B and C where mock transfectants were used.

### FYF-ITK Mutant is Deficient in its Ability to Become Phosphorylated

**Figure 3:**
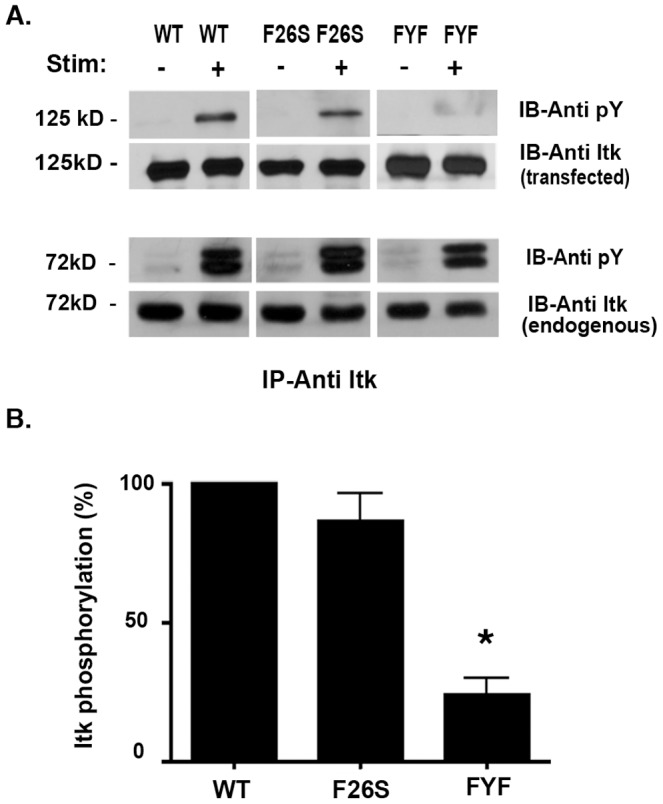
FYF-ITK mutant is deficient in its ability to become phosphorylated upon TCR stimulation. *(A)*, Jurkat cells transfected with cDNA constructs encoding CFP/YFP chimeric WT-, F26S- and FYF-ITK mutants were stimulated with anti-CD3ε (+) or isotype control (-) antibodies, lysed, and ITK (both transfected and endogenous) immuno-precipitated (IP) with anti-ITK antibodies. The immune complexes were resolved by SDS-PAGE, proteins transferred onto PVDF membranes, and immuno-blotted (IB) sequentially with anti-phosphotyrosine and anti-ITK antibodies as indicated. The upper sets of panels represent transfected and the lower sets endogenous ITK. Signals were developed by chemiluminescence. *(B)*, Bands from three replicate experiments (including the one displayed in panel A) performed as in (A) were quantified using ImageJ software and displayed as the percentage of transfected WT-ITK phosphorylation calculated as described in the materials and methods section. The * denotes that the difference between FYF-mutant and WT or F26S is statistically significant at p<0.05 determined by the student’s t test.

The enzymatic activation of ITK is dependent on the LCK-mediated phosphorylation of the C-terminal tyrosine 511 located in the activation loop of ITK [15]. To assess whether the impaired PLCγ_1_ activation in FYF-ITK mutant expressing cells reflects impaired ITK activation, we transfected Jurkat T cells with either FYF-mutant or WT-ITK constructs and then stimulated them with anti-TCR antibodies. Compared to the WT-ITK controls, FYF-ITK mutant had reduced phosphorylation (Fig. 3A, top panels). In contrast, mutation of only one of the residues in the FYF motif (F26S) displayed no defect in phosphorylation (Fig. 3A, top panels). This was true for all single mutations in the FYF motif (not shown). The defect in phosphorylation was specific to FYF-ITK mutant because endogenous ITK was phosphorylated normally in the same cells (Fig. 3A, lower panels). The defective phosphorylation of FYF-ITK mutant was reproducible in three replicate experiments, revealing an average of seventy-five percent reduction in the phosphorylation of the FYF-ITK mutant (Fig. 3B). The strong dependence of endogenous WT-ITK phosphorylation on induction by TCR stimulation (Fig. 3A, lower panels) and the differential effects on WT vs. FYF-mutant ectopic ITK expressed at similar levels (upper panels) indicate that the PTEN/SHIP1-deficiency of Jurkat cells does not confound the result that FYF-ITK mutant has a selective activation defect compared to WT-ITK.

### FYF-ITK Mutant Displays Intact Interactions with its Signaling Partners

**Figure 4:**
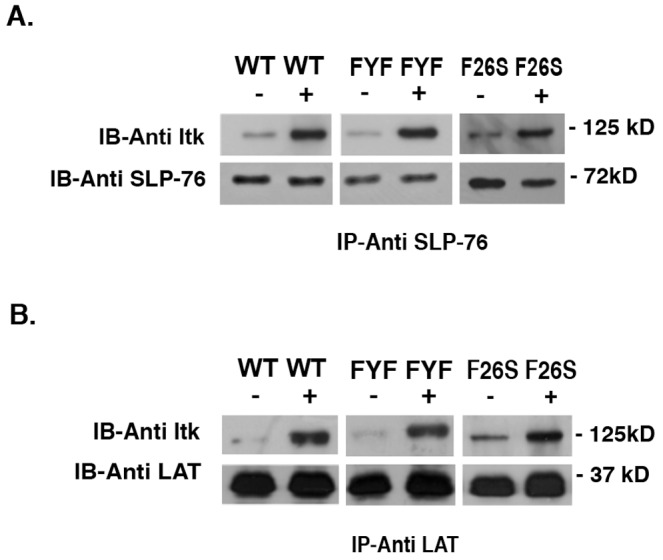
TCR-induced association of FYF-ITK mutant with SLP-76 and LAT is intact. *(A),* Jurkat cells transfected with the indicated CFP/YFP chimeric ITK mutant constructs were stimulated with anti-CD3ε or isotype control antibodies, lysed, and immunoprecipitated (IP) with anti-SLP-76 antibodies. Immune complexes were resolved by PAGE, proteins transferred onto PVDF membranes, and immunoblotted (IB) sequentially with anti-ITK and anti-SLP-76 antibodies (loading control) as indicated. *(B)*, lysates of cells similarly transfected, stimulated, and lysed were immunoprecipitated with anti-LAT antibodies and immune complexes resolved and sequentially immunoblotted with anti-ITK and anti-LAT antibodies. Bands were visualized by chemiluminescence as described in Materials and Methods section. Results are representative of three replicate experiments with the exception of F26S that represents a single experiment.

ITK phosphorylation and activation depend on its interaction with at least two intracellular signal transducers, SLP-76 and LAT (reviewed in ref. [2]). To determine whether the defective FYF-ITK mutant activation reflects impaired SLP-76/LAT interactions, we assessed the association of FYF-ITK mutant with these two adaptor proteins. Jurkat T cells that had been transfected with either FYF-mutant or WT ITK were stimulated through the TCR and lysates were subjected to co-immunoprecipitation with antibodies to SLP-76 and LAT. Interestingly, FYF-ITK mutant displays normal basal and TCR-induced association with these two adaptors that was similar to WT-ITK and F26S-ITK proteins (Fig. 4A, B). Therefore, the defective activation we see with FYF-ITK mutant cannot be explained by defective interactions with these two signaling partners.

### ITK-FYF Mutant Fails to Bind PIP_3_

**Figure 5:**
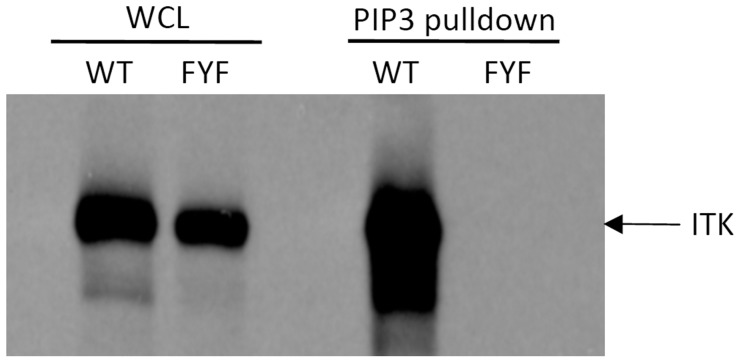
FYF-mutant ITK fails to bind PIP_3_. Whole cell lysates (WCL) obtained from HEK-293T cells transfected with untagged WT- or FYF-ITK mutant were resolved by SDS-PAGE and analyzed by immunoblotting with anti-ITK antibodies (left panels). An aliquot of each lysate was incubated with PIP_3_-coated beads followed by washing, elution, and SDS-PAGE/immunoblot analysis for ITK content (right panels). Results are representative of three independent experiments.

The recruitment of ITK to the cell membrane and its localization to the T cell-APC contact site are regulated through the production and turnover of the membrane lipid PIP_3_, which binds to the ITK PH domain [4]–[6]. Since the amino acids comprising the FYF motif may be important for the binding of membrane phospholipids to the PH domain, we tested the ability of FYF-ITK mutant to bind PIP_3_. To this end, HEK-293T cells were transfected with either FYF-mutant or WT ITK and lysates containing the expressed proteins were tested for their ability to bind the phospholipid by incubation with PIP_3_-coated beads and analysis of bound ITK as previously described [5]. The data shown (Fig. 5) clearly demonstrate that in contrast to WT-ITK, similar amounts of FYF-ITK mutant completely lack the ability to bind PIP_3_. Even at longer exposures of the immunoblot, there was no evidence of FYF-ITK mutant binding (not shown). These data indicate that the FYF motif of ITK is critical for the ability of PIP_3_ to engage the PH domain of ITK.

### TCR-induced Localization of FYF-ITK Mutant to the T Cell-APC Contact Site

**Figure 6:**
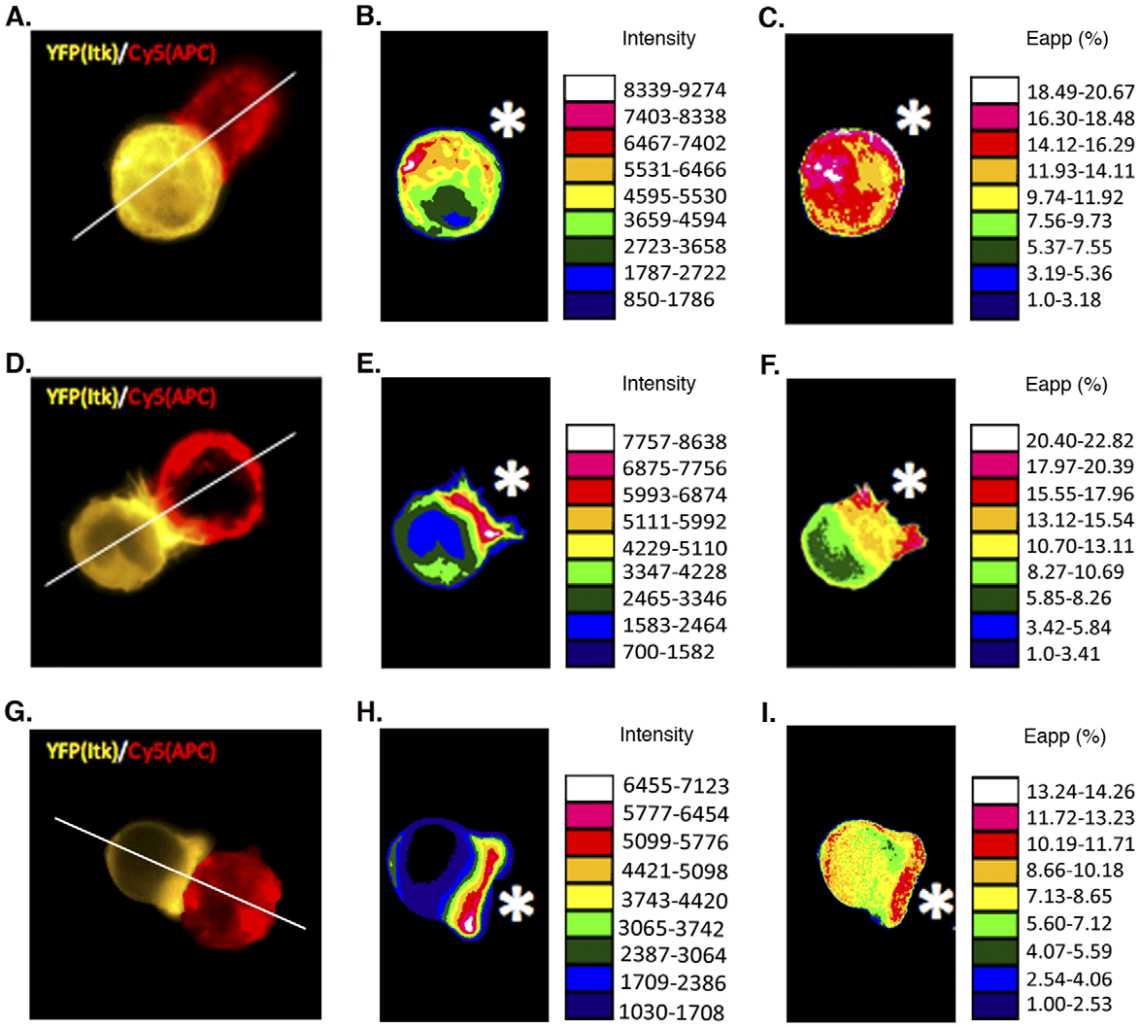
TCR-induced localization and conformational changes of ITK at the T cell-APC contact site. Jurkat cells that had been transfected with CFP/YFP chimeric constructs of WT- or FYF- ITK were incubated with SEE-pretreated Raji cells (labeled with Cy5 ester) and then fixed on slides and analyzed by epifluorescence microscopy for ITK localization and FRET as described in the Materials and Methods section. *(A),* representative example of conjugate between Jurkat transfected with WT-ITK (yellow) and SEE-pretreated Raji cell (red) displaying ITK localization under no-stimulation (3 min at 4°C) conditions. *(B),* the Jurkat cell in (A) presented as a pseudocolored intensity map with look-up table. *(C)*, the same cell analyzed for FRET and presented as a pseudocolored intensity map of E_app_ (%) with look-up table. *(D)*, representative example of conjugate between Jurkat transfected with WT-ITK and SEE-pretreated Raji displaying ITK localization under stimulation (3 min at 37°C) conditions. *(E)*, the Jurkat cell in (D) presented as a pseudocolored intensity map with look-up table. *(F)*, the same cell analyzed for FRET and presented as a pseudocolored intensity map of E_app_ (%) with look-up table. *(G)*, representative example of conjugate between Jurkat transfected with FYF-ITK and SEE-pretreated Raji displaying ITK localization under stimulation (3 min at 37°C) conditions. *(H)*, the Jurkat cell in (G) presented as a pseudocolored intensity map with look-up table. *(I)*, the same cell analyzed for FRET and presented as a pseudocolored intensity map of E_app_ (%) with look-up table. Images of FYF-ITK transfected Jurkat under non-stimulation conditions are identical to the image shown in panel A (data not shown). White stars in the intensity map panels represent the relative position of Raji cells.

**Figure 7:**
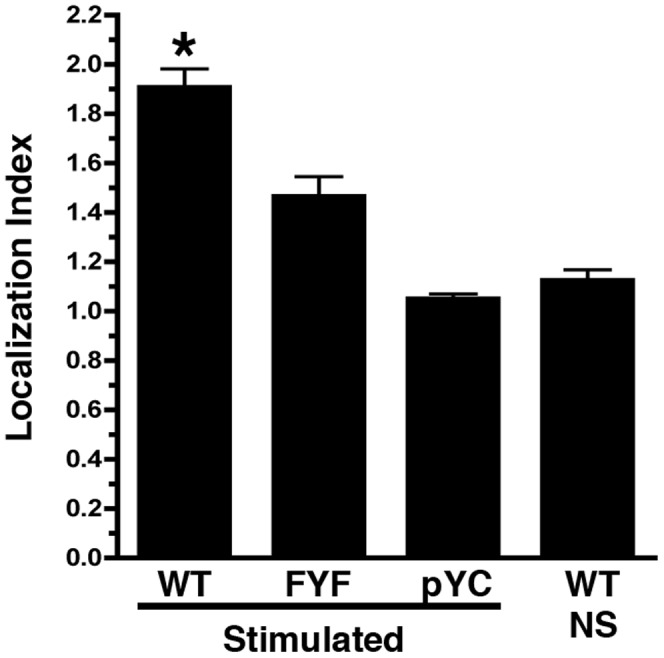
Cumulative localization data of ITK at the T cell-APC contact site. Conjugates between Raji and Jurkat cells transfected with WT-ITK, FYF-ITK, or pYC (construct containing only the two fluorescent proteins separated by a short linker) were treated as in Figure 6 (A, D, G) and ITK localization was assessed as described in Materials and Methods. Results are displayed as the average Localization Index (±SEM) for each transfectant calculated as described in the Materials and Methods section. WT/NS denotes non-stimulation conditions as in Figure 6A. The number of conjugates analyzed in each group are WT, N = 129; FYF, N = 55; pYC, N = 21; WT/NS, N = 10. The * indicates p<0.05 between WT and the rest of the groups determined by the student’s t test. FYF-ITK is also significantly different from pYC at p<0.05.

The activation of T cells through the TCR induces the intracellular redistribution of ITK from the cytoplasm to the T cell-APC contact site and its association with the TCR complex [4], [5]. In order to determine the ability of FYF-ITK mutant to become recruited to the T cell-APC contact site, we created chimeric ITK constructs where the fluorescent proteins CFP and YFP were coupled to the N- and C-terminal ends of the kinase, respectively. Use of such double chimeric constructs and the FRET technique enables the assessment of ITK subcellular localization, as well as potential conformational changes that a given mutation may produce (see below). Representative examples of the intracellular distribution of WT-ITK under resting and TCR-induced activation conditions are shown in Figures 6A and D respectively. Under resting conditions ITK is diffusely distributed in the cytoplasm (Fig. 6A, yellow pixels), but upon stimulation through a SEE-treated surrogate antigen-presenting cell (APC; Raji-SEE) ITK is redistributed and localizes to the T cell-APC contact site (Fig. 6D; yellow pixels at contact site). Interestingly, even though the localization of FYF-ITK appears seemingly unaffected -if one visualizes the single conjugate presented in Figure 6G- a quantitative assessment of localization at the T cell-APC contact site and determination of a localization index (described in Materials and Methods), reveals a 24% reduction in the localization of FYF-ITK mutant as compared to the WT-ITK control (Fig. 7). This reduction is statistically significant at p<0.05 for the number of conjugates compared (WT, 129 and FYF, 55 conjugates). Localization to the contact site is specific for ITK because an empty vector construct (pYC) containing just the two fluorophores did not enrich at contact sites (Localization Index of 1; Fig. 7). The difference of the Localization Index between FYF-ITK and pYC is significant at p<0.05.

**Figure 8:**
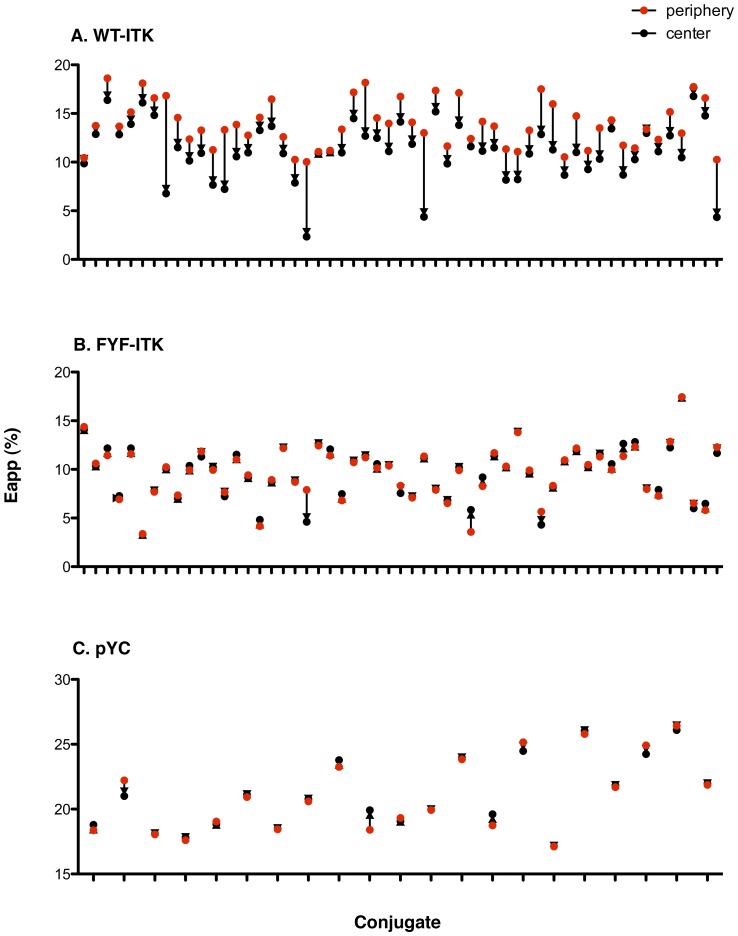
TCR-induced changes in E_app_ at the T cell-APC contact site. *(A)*, Jurkat T cells transfected with WT-ITK were incubated with SEE-pretreated Raji cells and E_app_ at the center and periphery (average of right and left sides) of the contact site was assessed as described in the Materials and Methods section. Red and black dots represent the E_app_ at the periphery and center of the same conjugate, respectively. The values displayed are those of 55 representative conjugates out of 114 total. Differences in E_app_ at the periphery and center of the contact of each individual conjugate are significant (p<0.0001, paired student’s t test). *(B),* Jurkat cells transfected with FYF-ITK and treated as in (A). The values of 55 conjugates are displayed. Differences in E_app_ at the periphery and center of the contact of each individual conjugate are not significant (p = 0.5973, paired student’s t test). *(C),* Jurkat cells transfected with pYC control (construct containing only the two fluorescent proteins separated by a short linker) and treated as in (A). The values of 21 conjugates are displayed. Differences in E_app_ at the periphery and center of the contact of each individual conjugate are not significant (p = 0.6366, paired student’s t test).

FRET has been previously used to assess conformational changes in intracellular proteins of lymphocytes upon activation through the TCR molecular complex [16]. If TCR-induced activation were to result in a conformational change on ITK, we would expect to see a change in the apparent efficiency of FRET (E_app_) between the N-terminus-attached CFP donor and the C-terminus-attached YFP acceptor. To this end, we visualized the intracellular patterns of FRET in CFP-ITK-YFP-expressing T cells in response to TCR activation by superantigen (SEE)-loaded surrogate APC. The CFP-ITK-YFP sensor exhibited intermediate FRET (E_app_∼9%) that was lower from the FRET (E_app_∼21%) in the directly coupled CFP-YFP (pYC) construct, thus enabling sensitive detection of any conformational changes that would alter the N-C termini distance and/or orientation. We observed a rather randomly distributed FRET (E_app_) pattern across the entire cell without stimulation (Fig. 6C). In contrast, a very distinctive E_app_ pattern was seen upon TCR stimulation (Fig. 6F). In this pattern E_app_ was relatively higher at the periphery of the contact site and lower at its center (see representative conjugate in Fig. 6F and compare to 6C). In order to verify the significance of this activation-induced spectral change, we compared FRET values at the center of contact for each of 114 individual conjugates to their respective FRET at the periphery (average of right and left) of the contact. There was a significant reduction in FRET at the center of each contact compared to the periphery of the same contact at p<0.0001 as assessed by the paired student’s t test (Fig. 8A). For the sake of clarity we only show 55 representative conjugates out of the 114 analyzed. In sharp contrast, such changes in the FRET pattern under activation conditions were not seen with FYF-ITK (see representative conjugate in Fig. 6I and compare to 6F). Analysis of 55 such conjugates reveals no significant differences in E_app_ (Fig. 8B, p = 0.5973). This observation was specific for ITK because a pYC construct containing only the fluorophore proteins did not display these changes (Fig. 8C, p = 0.6366). The data presented in Figure 8 argue against these changes being due to random changes in the orientation of the donor and acceptor fluorophores. If this were the case one would not expect to see the reduction in FRET limited only to WT-ITK. Thus, even though the FYF mutation only partially impairs ITK recruitment to the contact site (Fig. 7), those mutant molecules that do localize to the contact site fail to assume the specific TCR-induced conformational changes seen with WT-ITK (Fig. 8 A and B).

## Discussion

The *Tec* family of protein tyrosine kinases is unique in that its members (with the exception of RLK) possess PH domains at their N-termini that interact with membrane phospholipids [17]. PH domains play a significant role in the inducible recruitment of *Tec* kinases to the plasma membrane and the regulation of their activation [17]. Although PH domains share only limited sequence homology, their three-dimensional structures are strikingly similar and are composed of a β-barrel made of seven β-strands that form two perpendicular β-sheets followed by a C-terminal α-helix [18]. In 10–20% of the approximately 300 mammalian PH domains, this conserved β-barrel structure contains a binding pocket for membrane phospholipids [19]. In the case of the *Tec* kinases ITK, BTK, and TEC, a membrane phospholipid that binds their PH domains with high affinity and specificity is the phosphoinositide 3-kinase (PI3K) product phosphatidylinositol 3,4,5-trisphosphate (PIP_3_; ref. [20]). Consequently, recruitment and activation of these kinases are likely to be controlled by PIP_3_ generation and turnover by PI3K or the SHIP/PTEN phosphatases, respectively. Interestingly, a recent report from one of our labs has demonstrated that the soluble PH domain ligand, inositol 1,3,4,5-tetrakisphosphate (IP_4_), regulates the binding of PIP_3_ to the PH domain of ITK and TEC [5].

Three aromatic residues, termed the FYF motif, located in the inner walls of the phospholipid-binding pocket, are conserved in the PH domains of all *Tec* kinases [9]. The fact that these three amino acids are not conserved in the PH-domains of non-*Tec* proteins suggests that the FYF motif might be involved in a specific function of *Tec* kinases [9]. It has been suggested that in BTK, the amino acids of its FYF motif (F25, Y112, F114) might be critical for membrane phospholipid binding. Their mutation is suspected to cause X-linked agammaglobulinemia [9]. In fact, a mutation in one of these residues (F25S) has been uncovered in a patient with X-linked agammaglobulinemia [21].

The biological significance of the ITK FYF motif, located in amino acids F26, Y90, and F92, has not been previously addressed. Therefore, to assess its importance, we constructed a triple mutant (F26S, Y90F, F92S) cDNA, henceforth termed FYF-ITK mutant, and tested its impact on the function of ITK. Upon expression of the FYF-ITK mutant in thymocytes from ITK^−/−^ mice, we found that the TCR-induced production of the Th_2_ signature cytokine IL-4 was significantly reduced (Fig. 1). Since there is no evidence that ITK regulates Th_2_ cytokines selectively, and the fact that some of the Th_2_ cytokine genes (including IL-4) are clustered [22], it is very likely that the FYF mutation has similar effects on other Th_2_ cytokines. In contrast to WT-ITK, the FYF mutant failed to rescue the inability of ITK-deficient cells to phosphorylate PLC-γ_1_ on its Tyr 783 (Fig. 2). This is relevant because production of IL-4 and other cytokines depends on the phosphorylation and activation of PLC-γ_1_ [13], and phosphorylation of Tyr 783 is one of the targets of ITK [14]. These observations were consistent with the fact that the phosphorylation (and thus, activation) of the FYF-ITK mutant was significantly reduced (Fig. 3), as was the ability of this triple mutant to bind PIP_3_ (Fig. 5).

Previous evidence has demonstrated that for TCR-induced activation of ITK, the ITK kinase must interact with a signalosome encompassing the adaptors LAT and SLP-76 [11], [13], [14], [23], [24]. Since previous studies indicate that LCK associates inducibly with LAT [25] and with SLP-76 [24], [26], it is reasonable to envision that interaction with this signalosome brings ITK into close proximity to LCK, which activates ITK by transphosphorylation on Tyr 511 of its activation loop [15]. Therefore, it was surprising to see that even though the FYF-ITK mutant was able to interact with these adaptor proteins (Fig. 4), it was not phosphorylated (Fig. 3). The possibility that the unimpaired interaction of the FYF-ITK mutant with the LAT/SLP-76 signalosome might be due to the overexpression of total ITK levels in Jurkat cells is unlikely. In previous studies from our lab, where the same experimental system was used, expression of other ITK mutants at similar levels to those seen here, have displayed differential effects on their inducible association with LAT and/or SLP-76 that depend on the particular mutation and not ITK expression levels [11], [27]. Thus, even though the association of ITK with the LAT/SLP-76 signalosome might be necessary for ITK activation, by itself it is not sufficient and other events must be involved. One such event could be the interaction of ITK with the membrane phospholipid PIP_3_, which has been shown to be critical for its activation [5]. It appears, therefore, that interaction with membrane phospholipids although not a pre-requisite for the association of ITK with the LAT/SLP-76 signalosome, it is important for ITK phosphorylation and activation.

The recruitment of the FYF-ITK mutant to the T cell-APC contact site, although significantly reduced, was not totally ablated (Fig. 7). However, our FRET analysis suggests that FYF-ITK molecules that did migrate to the contact site, displayed some undefined conformational differences compared to WT-ITK (Fig. 6F and I and Fig. 8). WT-ITK that localized inducibly at the periphery of the T cell-APC contact site displayed significantly higher FRET activity compared to ITK that localized at the center of the contact. In contrast, FYF-ITK did not display this behavior. This difference in FRET pattern seen with the WT-ITK implies some sort of conformational change in the ITK molecule that presumably does not occur due to the FYF mutations. The nature of this conformational change and how it may affect other events at the contact site are not understood. It is interesting that recent studies by Singleton et al have demonstrated that ITK recruited to the center of the contact site controls the spatiotemporal organization of other signaling molecules that are recruited to the site upon TCR engagement [28]. Thus, it would be interesting to know how the FYF mutations would affect these spatiotemporal arrangements.

Another interpretation of the contrasting effects of the FYF mutations on PIP_3_ binding and association with the LAT/SLP-76 complex may relate to the observations that ITK must undergo intermolecular self-associations in order to become activated [29]. Upon TCR activation, cytoplasmic ITK is recruited to the cell membrane where it forms dimers and/or higher order multimers that are PH domain dependent and may be regulated by membrane phospholipids [5], [29]. Thus, it is attractive to hypothesize that perturbations in PIP_3_ binding, such as the one seen with the FYF mutant, alters these PH domain-dependent self-associations and impairs ITK activation, but surprisingly not the ability of ITK to interact with the LAT/SLP-76 complex.

Another mechanism of TCR-induced ITK intermolecular self-associations involves the interaction between the SH2 domain of one ITK molecule with the SH3 domain of another ITK molecule [30]. This SH2-SH3 interaction is non-canonical to the extent that these domains interact with each other via amino acid residues distinct from those that recognize their classical ligands (SH3-polyproline and SH2-phosphotyrosines; ref. [31]). In recent experiments, we observed that disruption of this type of ITK self-associations does not affect the recruitment of ITK to the T cell-APC contact site, but it interferes with the ability of ITK to interact with the SLP-76 signalosome [27]. Thus, distinct ITK domain mediated interactions that differentially regulate the recruitment of ITK to the T cells-APC contact site and its association with the LAT/SLP-76 signaling complex appear to regulate TCR-induced activation of this kinase.

Another mutation in the PH domain of ITK that causes defects in PIP_3_ binding involves the substitution of arginine 29 with cysteine (R29C; ref. [5]). It is not known, however, whether the R29C mutant becomes inducibly recruited to the T cell-APC contact site or whether its association with the LAT/SLP-76 signalosome is affected. Studies on the structure of the BTK PH-domain have shown that the analogous amino acid (R28), located close to the first phenylalanine of the FYF motif, is at the border of the phospholipid-binding site and its mutation (R28C) also disrupts PIP_3_ binding [9]. Thus, it is very likely that R29C and FYF in the ITK PH-domain may represent a tetrad of amino acids on the inner walls of the PH-domain β-barrel that form an important component of the PIP_3_ binding site. This is supported by the fact that the FYF mutant was unable to bind PIP_3_ (Fig. 5).

The possibility that lack of PIP_3_ binding by the PH domain of the FYF-ITK mutant is due to structural disorganization of the domain due to the triple mutation –a situation that would mimic a PH domain deletion mutant (ΔPH)- is highly unlikely. Evidence that argues against this possibility is the fact that the FYF-ITK mutant does localize to the T cell-APC contact site, albeit at significantly lower levels compared to WT-ITK (Fig. 7). If FYF-PH domain is structurally disorganized, we would expect that the FYF mutant would not be able to localize at all, as observed for the ΔPH-ITK mutant [4]. In addition, previous studies have demonstrated that ΔPH-ITK displays very high spontaneous enzymatic activity [4], an event that has not been observed with the FYF-ITK mutant. Finally, another possibility might be that FYF-ITK is not recruited to the “proper” site in the membrane and thus it is unable to become activated upon TCR stimulation.

The data presented here reveal a novel set of amino acids in the PH domain of ITK that are critical for its inducible activation by the TCR. These amino acids termed the FYF motif appear to be critical for the binding of the membrane phospholipid PIP_3_ to the PH domain. Even though their mutation does not completely inhibit the recruitment of ITK to the T cell-APC contact site, it appears to cause conformational changes in ITK that might be important in the intermolecular self-associations of ITK and its subsequent activation. Interestingly, in contrast to the requirement for membrane phospholipid binding, the FYF motif is dispensable for adaptor protein interactions. Along with previously published reports, the present investigation adds to the evidence for multiple signaling events that regulate the TCR-mediated activation of ITK.

## Materials and Methods

### Ethics Statement

All protocols using mice were approved by the IACUC of San Diego State University. Animal Welfare Assurance Number A3728-01.

### Mice

Mice deficient in ITK expression (ITK^−/−^) were obtained from Dr. D. Littman (New York University School of Medicine) and bred in our own animal facility. These mice have been previously described [32]. Male or female mice of 6–12 weeks old were used as source of thymocytes in nucleofection experiments.

### Cell Lines

The JTag subline of Jurkat cells was a kind gift of Dr. A. Altman (La Lolla Institute of Allergy and Immunology). This cell line has been previously described [33]. Raji (CCL-86) and HEK-293T cells (CRL-11269) were obtained from the American Type Culture Collection. The former cell line was cultured in RPMI 1640 (Mediatech) and the latter in DMEM (Mediatech) medium both supplemented with 10%FBS (SAFC Biosciences), 2 mM L-Glutamine, 100 U/ml penicillin, 100 µg/ml streptomycin, 20 mM HEPES (all obtained from Mediatech) at 37°C in a humidified atmosphere of 5% CO_2_-air.

### Antibodies and other Reagents

Anti-human CD3ε monoclonal antibody (OKT3) was produced in-house from hybridoma CRL8001 obtained from ATCC. Anti-mouse CD3ε (2C11, cat# 553058), anti-mouse CD28 (37.51, cat# 553295), and Alexa Fluor 647-conjugated anti-PLCγ1 pY783 (27/PLC, cat# 557883) antibodies were purchased from BD Pharmingen. Rabbit polyclonal anti-ITK antibody (cat# 06-546) for immunoprecipitation, mouse monoclonal anti-ITK antibody (2F12, cat# 06-476) for immunoblotting, sheep anti-SLP-76 antibody (cat# 06-548) for immunoprecipitation, anti-LAT for both immunoprecipitation and immunoblotting (clone 2E9, cat# 05-561), and anti-phosphotyrosine antibody (4G10, cat# 05-321 ) were obtained from Millipore/Upstate. Rabbit anti-SLP-76 antibody (cat# 4958) used for immunoblotting was purchased from Cell Signaling Technology. Other antibodies used were DyLight 405-conjugated anti-Golden Syrian and Armenian Hamster antibody mix (cat# 620-146-440) from Rockland Immunochemicals, goat anti-Armenian Hamster (cat# 127-005-160) and rabbit anti-mouse IgG (catalog number 315-005-044) from Jackson ImmunoResearch, isotype control antibody UPC-10 (cat# M5409) from Sigma Aldrich. Recombinant protein G-Sepharose (code# 17-0618-01) was purchased from GE Healthcare.

### cDNA Constructs, Transfection and Nucleofection

FYF-ITK mutant, K390R-ITK and single point mutants F26S-, Y90F-, F92S-ITK were generated using the GeneTailor site directed mutagenesis kit (Invitrogen) and subcloned and expressed into the pME18s vector for transfection into Jurkat and 293T cells after verification by sequencing. In these transfection experiments chimeras of the mutant constructs, as well as WT-ITK, were used with CFP and YFP at the N- and C-termini respectively. Jurkat transfections were performed by electroporation using the Gene Pulser (BioRad) whereas 293T cells were chemically transfected using Lipofectamine 2000 (cat# 11668-019; from Invitrogen). All of the above procedures have been previously described [4]. Primary mouse thymocytes, isolated from the thymi of ITK-deficient mice by separation through a nylon wool mesh, were nucleofected with endotoxin-free preparations of GFP chimeras of the above constructs subcloned into a pCMV-driven vector (Clontech). For nucleofection, we used the Amaxa Nucleofector II (program X-001) following the manufacturer’s recommendations (Lonza). The nucleofected cells were cultured in 1.5 ml of Lonza Nucleofection medium supplemented with 10% FBS at 37°C in a humidified 5% CO_2_-air atmosphere for 16–24 hours before used in experiments.

### Skewing, Interleukin-4 Production, and Assay

To increase the frequency of Interleukin-4 (IL-4) producing T cells, thymocytes that had been nucleofected with cDNA constructs described above were cultured under Th_2_ skewing conditions using a modification of previously published protocols [34]–[36]. Following the nucleofection procedure described above, 2×10^5^ thymocytes re-suspended in 100 µl of Lonza medium (10% FBS) containing anti-CD28 (1 µg/ml) and anti-IL-12 (20 µg/ml) antibodies were cultured (37°C, 5% CO_2_-air) for 24 hours in round bottom wells of non-pyrogenic round-bottom 96-well microtiter plates (Sarstedt) that had been pre-coated with anti-CD3ε antibody 2C11 (1 µg/ml). Cultures were then supplemented with Interleukin-2 (5 ng/ml) and Interleukin-4 (10 ng/ml) and incubated for an additional 48 hours. Cells were then thoroughly washed and re-stimulated by transferring to new anti-CD3ε coated plate wells and culturing in medium containing anti-CD28 (1 µg/ml) antibody for 24 hours as above. Cell-free culture supernatants were assayed for IL-4 using an ELISA kit (BD Bioscience, cat# 555232) with sensitivity of detection at 8 pg/ml. Analysis was performed in duplicate on an eMax Precision Microplate Reader using SoftMax Pro v5.0 software (Molecular Devices).

### Thymocyte Stimulation and PLCγ_1_ Phosphorylation

TCR-induced intracellular phosphorylation of PLCγ_1_ was assessed by using a modification of previously published methods [37], [38]. Thymocytes nucleofected with GFP-chimeric constructs of WT-, K390R-, or FYF-mutant ITK were incubated for one hour on ice with 0.5 ml of medium containing Armenian Hamster anti-mouse CD3ε (1 µg/ml) antibody followed by cross-linking of the primary antibody. This involved pelleting and re-suspending the cells in 0.5 ml of ice-cold medium containing a pre-mixed cocktail of 7 µg goat anti-Armenian Hamster antibody and 0.5 µg of DyLight 405-conjugated mix of anti-Golden Syrian and Armenian Hamster antibodies and incubation for an additional 30 minutes. The inclusion of DyLight 405-conjugated antibody was used in order to identify the cells that bound the stimulating anti-CD3ε antibody (potentially stimulated cells). Stimulation was then initiated by incubating the cells in a 37°C water bath for 1 minute and immediately fixing them by adding an equal volume of ice-cold paraformaldehyde (2% final concentration for 10 min) and permeabilized by re-suspension in 1 ml of ice-cold methanol (100%) and storage overnight. After thorough washing (PBS-0.5% BSA) and blocking with 100 µg/ml of mouse IgG (1 hr-RT°) to minimize non-specific reactivity, cells were stained with Alexa 647-conjugated anti-PLCγ_1_ pY783 (1 hr, RT° in the dark) following the manufacturer’s recommendation. The phosphoflow signal was detected using a FACSAria flow cytometer (BD Biosciences) and the data analyzed with FlowJo 7 software (Tree Star). Negative controls included thymocytes that were either nucleofected or not and treated as above, but in the absence of anti-mouse CD3ε. These two controls revealed similar histograms. For flow cytometric analysis of the phosphoflow signal, a gate was first set encompassing GFP-positive (transfected) cells (mock-transfected cells were used as controls). The cells included in this gate were further gated to include the top 15% MFI of DyLight 405 signal (defined as stimulated cells). Within this gate we analyzed the Alexa 647 signal (pY783 specific). In the experiments shown, the average percentage (±SEM) of pY783 positive cells nucleofected with WT-ITK was 39.6% (±8.7). This value was set as 100% Y783 positive cells. The percentages in the ITK mutant nucleofected cells were standardized to this value.

### ITK Phosphorylation

This was performed by immunoprecipitation with anti-ITK and immunoblotting with anti-phosphotyrosine (4G10) antibodies as previously described [4], [24]. Percentage of transfected ITK phosphorylation was calculated by densitometric analysis of the immunoblotted bands using the NIH Image J software. One hundred percent of transfected WT-ITK phosphorylation was defined as the ratio of pixels detected in the phosphotyrosine band over the pixels of the respective ITK (transfected) loading control. The values of the mutant-transfected samples were standardized to these of the WT-ITK groups as percent of WT-ITK phosphorylation.

### PIP_3_ Binding Assay

PIP_3_ binding of ITK was analyzed as previously described [5]. Briefly, whole lysates from HEK-293T cells chemically transfected (Lipofectamine) with WT-ITK or FYF-ITK mutant were incubated with PIP_3_-coated beads for two hours, followed by SDS-PAGE and immunoblotting analysis with anti-ITK antibodies. Aliquots of the same lysates were included as loading controls.

### Jurkat-Raji Cell Conjugate Formation and ITK Localization

It was performed as previously described with some modifications [39]. Jurkat cells, 1×10^6^ in 200 µl, transfected with CFP/YFP chimeric ITK constructs as indicated, were incubated (3 minutes at 37°C or 4°C) with an equal number of Cy5-ester (Amersham-GE Healthcare) labeled Raji cells that had been pre-treated (2 hours−37°C) with 10 µg/ml of SEE (Toxin Technology). The cell mixture was then fixed with an equal volume of 4% paraformaldehyde, washed, and mounted on slides. Localization of ITK was assessed by epifluorescence microscopy using an AxioVision Imaging System (Carl Zeiss) with 0.5 neutral density filter. Conjugates were selected morphologically by Differential Interference Contrast (DIC) using the stable conjugate criteria established by Montoya et al [39]. The ITK localization index was calculated as we have previously described [24] using the NIH ImageJ software. Briefly, a transect was drawn across the T cell (see Figure 6A, D, and G) and the ratio of pixels of the ITK image at the contact site over those at the opposite site was defined as the ITK localization index.

### Imaging of CFP and YFP and Assessment of FRET

The Raji-Jurkat cell conjugates described above, where Jurkat cells were transfected with chimeric ITK constructs with CFP and YFP at the N- and C-termini respectively, were used for FRET imaging and analysis at the T cell-APC contact site as previously described [40], [41]. Image collection was performed with a Zeiss AxioVision Imaging System and the NIH ImageJ software with a custom-designed macro used for image analysis. Briefly, for each region of interest three exposure intensities (I) were acquired using a three-filter set (Chroma); CFP excitation/CFP emission (I_DD_), YFP excitation/YFP emission (I_AA_), and CFP excitation/YFP emission (I_DA_). Background was subtracted based on a cell-free region of each image and bleed-through coefficients of CFP into CY (FRET) channel (‘d’ parameter) and YFP into CY channel (‘a’ parameter) were determined using cells expressing CFP-ITK or YFP-ITK alone. To avoid pixel shift, images were aligned by vector-spline regularization using TurboReg plugin for ImageJ before analysis. FRET efficiency was calculated using the formula E_app_ = (F_c_)/(F_c_+G*I_DD_) where F_c_ = I_DA_ − a*I_AA_ − d*I_DD_ represents the sensitized acceptor emission (FRET) corrected for spectral bleed-through, and G (Gordon parameter) represents an instrument specific parameter that relates the level of sensitized emission to donor quenching [41]. In our set up, G = 5.151 was calibrated as the ratio of F_c_ to donor fluorescence recovery after acceptor photobleaching.

### Immunoprecipitation, Immunoblotting, PAGE, in vitro Kinase Assay, and Jurkat Cell Stimulation

All these techniques have been previously described in other publications by our laboratory [24].
